# The Potential Effects of Light Irradiance in Glaucoma and Photobiomodulation Therapy

**DOI:** 10.3390/bioengineering10020223

**Published:** 2023-02-07

**Authors:** Sang-Hyun Ahn, Jung-Soo Suh, Gah-Hyun Lim, Tae-Jin Kim

**Affiliations:** 1 Department of Integrated Biological Science, College of Natural Sciences, Pusan National University, Pusan 46241, Republic of Korea; 2 Department of Biological Sciences, College of Natural Sciences, Pusan National University, Pusan 46241, Republic of Korea; 3 Institute of Systems Biology, Pusan National University, Pusan 46241, Republic of Korea

**Keywords:** blue light, glaucoma, oxidative stress, photobiomodulation, retinal ganglion cell

## Abstract

Human vision is mediated by the retina, one of the most critical tissues in the central nervous system. Glaucoma is a complex retinal disease attributed to environmental, genetic, and stochastic factors, all of which contribute to its pathogenesis. Historically, glaucoma had been thought of primarily as a disease of the elderly; however, it is now becoming more problematic as the incidence rate increases among young individuals. In recent years, excessive light exposure has been suggested as contributing to the rise in glaucoma among the younger generation. Blue light induces mitochondrial apoptosis in retinal ganglion cells, causing optic damage; red light increases cytochrome c oxidase activity in the electron transport system, reducing inflammation and increasing antioxidant reactions to promote cell regeneration. In conclusion, the minimization of blue light exposure and the general application of red light treatment strategies are anticipated to show synergistic effects with existing treatments for retinal disease and glaucoma and should be considered a necessary prospect for the future. This review introduces the recent studies that support the relationship between light exposure and the onset of glaucoma and discusses new treatments, such as photobiomodulation therapy.

## 1. Introduction

Given the development of modern technology and the changes in the lifestyle of modern society, reliance on electronic displays has been recognized as the main cause of the rise in the incidence of eye diseases. In particular, glaucoma, an age-related disease, has become a serious problem because its incidence rate continues to rise even in young people. Glaucoma is a neurodegenerative disease characterized by progressive and functional deterioration of the optic nerve head (ONH) and retinal nerve fiber layer (RNFL) [1]. Once the light reaches the retina, it is converted into electrical signals by photoreceptors, and the optic nerve transmits it to the brain [2]. Glaucoma causes the field of view to become narrower due to a reduction in the optic nerve cells, and as it worsens, it leads to blindness. By 2013, 64.3 million cases of glaucoma had been diagnosed worldwide. This number had increased to 76.0 million by 2020, and is projected to increase to 111.8 million by 2040 [3].

There are three main causes of glaucoma: age, ischemia, and structural factors. An increase in age increases the incidence of glaucoma due to cellular senescence and functional cell deterioration [4]. In addition, when the ocular perfusion pressure decreases due to hypotension, which is a representative symptom of ischemia, or ischemia occurs due to high blood pressure or diabetes, oxygen and nutrients are not smoothly supplied to the optic nerve, and damage to the optic nerve may occur. A leading cause of the structural factors is an increase in intraocular pressure (IOP), defined as the ratio of the discharged aqueous humor to the aqueous humor produced by the ciliary body [4,5]. Eventually, when the intraocular water discharge is suppressed due to the trabecular meshwork or damage to the iris, IOP increases. Depending on the cause (trabecular meshwork or iris), it is called primary open-angle glaucoma (POAG) or primary angle-closure glaucoma (PACG). However, glaucoma can occur even when the IOP is within the statistically normal range (10–21 mm Hg), which is classified as normal-tension glaucoma (NTG). NTG may be caused by secondary glaucoma [5], which, in turn, is caused by other eye diseases, such as neovascularization and uveitis, or differences in individual sensitivity to IOP. In particular, it has been reported that the incidence of glaucoma in patients with high myopia was much higher than in those without myopia due to structural abnormalities of the eyeball [6,7]. High myopia is a disease in which the axial length of the eyeball grows excessively, and the image is formed in front of the retina. Individuals with high myopia are structurally more susceptible to glaucoma than those without [8]. This supports the results of some epidemiological investigations that state that an increase in the number of highly myopic patients in the young and old due to modern lifestyle habits can lead to an increase in the incidence of glaucoma [6,9].

Glaucoma is not only increasing in incidence rate but also decreasing in the age of onset. According to an investigation by the Korean Health Insurance Review and Assessment Service, the number of glaucoma patients aged between 10–29 years continued to increase between 2013–2018. It has emerged as one of the main eye diseases threatening the quality of life [9]. The increasing prevalence of glaucoma in young people has several causes. Specifically, the increase in metabolic syndrome in young people due to the changes in eating habits in modern society has impacted the incidence of glaucoma [10,11]. Metabolic syndrome refers to a person suffering from three or more metabolic disorders, such as high blood pressure, diabetes, and high blood cholesterol. Lack of exercise, which is effective in preventing certain types of degenerative vision loss, has also contributed to the increase in the number of glaucoma patients [12,13]. In addition, excessive use of digital media (computers, mobile phones, etc.) increases sleep disturbance and insomnia, increases IOP due to incorrect posture or cortisol production disorders, and oxygen deficiency due to sleep apnea directly damages the optic nerve, leading to glaucoma [14]. Generations with high digital media consumption suffer from dry eye syndrome daily, which has a significant effect on the increase in intraocular pressure. Also, rubbing the eye and angiogenesis caused by dry eye syndrome directly damage the eye, causing glaucoma [15,16,17]. Among them, an increase in the number of patients with high myopia and increased exposure to blue light, effects of the modern lifestyle that depend on electronic displays, are drawing attention as the main causes [18,19,20,21,22]. Therefore, in this review, we focus on light exposure as the cause of glaucoma and summarize research trends and related content on glaucoma and light irradiance. In addition, we introduce the clinical effect of photobiomodulation (PBM) treatment, which has been of interest in recent years and provide essential data for comprehensively understanding the pathological mechanism of glaucoma according to the wavelength of light and for establishing a glaucoma treatment strategy.

## 2. Human Eye Anatomy and Physiology

**Figure 1:**
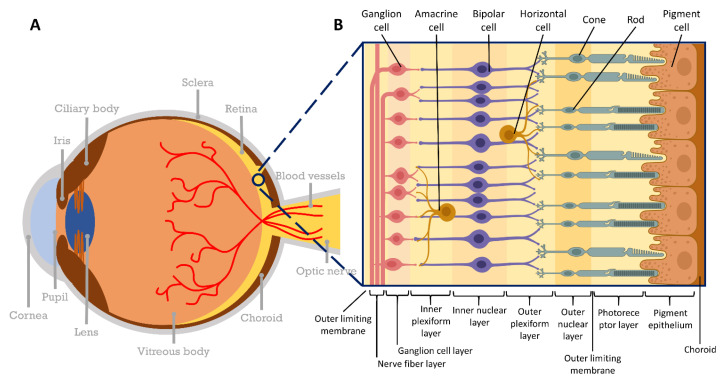
Structure of the eye and retina. (**A**) Structure of the eyeball consists of the cornea, lens, iris, macula, retina, and optic nerve that transmits visual information from the retina to the brain. (**B**) Cellular unit of the retinal layer. This figure was created using PowerPoint & BioRender (Available online: http://biorender.com (accessed on 9 April 2020)).

The normal human eye is a sphere that measures 24 mm in diameter and has unique anatomical and physiological functions [23]. There are three layers of membranes in the eye: the outer, the middle, and the inner layers, apart from other interior contents [24] (Figure 1A). The outer layer constitutes the cornea and the sclera; the middle layer, the choroid, the ciliary membrane, and the iris; and the inner layer, the retina, the lens, and the vitreous and aqueous humors. The retina is the tissue that lines the inner surface of the eye. The cells of the neural retina are composed of several parallel layers [25,26,27]. Several types of cells are found in the retina, such as photoreceptors, astrocytes, Müller cells, retinal ganglion cells, glial cells, amacrine cells, bipolar cells, horizontal cells, and retinal pigment epithelial (RPE) cells (Figure 1B). The nuclei of photo-receptors are located in the outer nuclear layer, and the outer segments are located proximally from the nuclei close to the RPE cells. The nuclei of Müller cells, bipolar cells, and amacrine cells are located in the inner nuclear layer of the retina. Bipolar cells and horizontal cells connect with photo-receptors, and bipolar cells and amacrine cells synapse with ganglion cells. The nuclei of retinal ganglion cells are in the ganglion layer, and their axons are in the nerve fiber layer. Müller cells also form synapses with dendrites of neurons and axons of the nerve fiber layer [25]. A part of the central nervous system, the retina converts light energy into electrical signals and transmits them to the brain via the optic nerve. In addition, the retina receives oxygen and nutrients through the retinal blood vessels and the choroidal capillaries in contact with Müller cells. RPE cells are an epithelial cell monolayer between photo-receptors and a layer of capillaries adjacent to the innermost layer of the choroid [26]. RPE cells consist of approximately 3.5 million epithelial cells arranged in a hexagonal pattern and relatively evenly distributed throughout the retina. Numerous pigments (melanin and lipofuscin) are present in the cytoplasm of RPE cells. Important functions of RPE cells include maintenance of photoreceptor function, adhesion to the retina, production of growth factors necessary for surrounding tissues, and wound healing upon injury [26,28,29,30,31]. In addition, it plays an important role in blood-retinal barrier function and metabolite excretion [32]. Glaucoma can develop due to aging, ischemia, or structural factors; these lead to optic disc cupping, a specific phenomenon of damage to the retinal nerve fiber layer (stratum opticum). The optic nerve disc concavity is a phenomenon in which the neuroretinal rim decreases in size due to an increase in the ratio of the optic cup to the optic disc and results in the deformation of the lamina cribrosa. As a result of the deformation of the lamina cribrosa, gradual damage to the axons and cell bodies of the retinal ganglion cells occurs. Therefore, when biochemical alterations in the optic nerve cause a decrease in blood flow and an increase in oxidative stress, excitatory toxicity, autophagy, apoptosis, and necroptosis-associated signal transduction processes occur. This damages the retinal ganglion cells and causes vision loss. A study by Weinreb and Khaw (2004) showed that a tomographic image of the retina could be observed in three dimensions using near-infrared rays through optical coherence tomography. In this study, the retinal nerve fiber layer and the ganglion cell inner plexiform layer (GCIPL) were examined in normal and glaucomatous eyes. GCIPL comparison was carried out to confirm the death of the retinal ganglion cells [1].

## 3. The Effects of Blue Light on the Eyes

**Figure 2:**
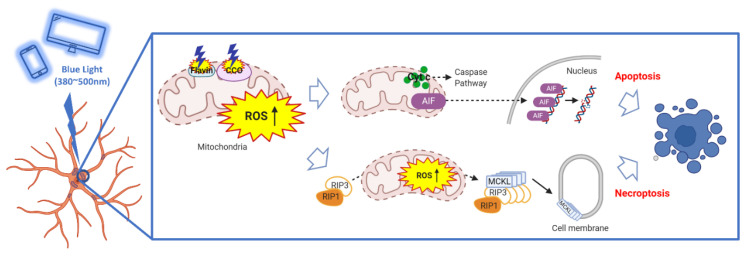
Schematic illustration of the proposed mechanism by which blue light causes glaucoma. This figure was created using PowerPoint & BioRender (Available online: http://biorender.com (accessed on 9 April 2020).

Light is necessary for the retina to convert light energy into electrical signals and transmit them to the brain via the optic nerve, even though it is not always beneficial to the retina. Several mitochondria are present in the nodes of Ranvier of retinal ganglion cells in the retina, which take up a significant amount of energy. Osborne et al. (2006) confirmed that blue light adversely affects the mitochondria of retinal ganglion cells. Moreover, there is evidence that the mitochondrial electron transport chain-related enzymes flavin and cytochrome C oxidase (CCO) are damaged by blue light, resulting in the generation of photochemical effects and reactive oxygen species (ROS) (Figure 2) [33]. ROS are normally regulated by antioxidants, but in eyes deformed by ischemia or myopia, blue light leads to excessive production of ROS and mitochondrial DNA damage. Ultimately, this results in the loss of the visual field owing to a cascade of events leading to cell death [22]. A previous study has shown that when exposed to blue light under ischemic conditions, the retina produces relatively low levels of ATP, the retinal ganglion cells are damaged, and mitochondrial energy metabolism is inhibited [34]. 

Despite the limitations associated with its visual assessment, it is possible to determine the degree of aging in cells by measuring the amount of galactosidase that accumulates with the progression of cellular aging and by detecting ROS inside the cells. Kernt et al. (2012), in an experiment using human retinal epithelial cells stained for detecting beta-galactosidase activity, showed that blue light-induced cell senescence and that the degree of intracellular ROS decreased when the blue light was filtered [19]. In another study, the 3-(4,5-dimethylthiazol-2-yl)-2,5-diphenyltetrazolium bromide (MTT) assay was used to confirm that the survival rate of retinal epithelial cells exposed to blue light was lower than that of cells exposed to darkness, suggesting that blue light induced a decrease in mitochondrial activity and lowered energy levels, resulting in the induction of apoptosis [18]. There are two general mechanisms by which programmed cell death is induced: apoptosis and necroptosis. Apoptosis is induced when the apoptosis-inducing factor (AIF), which exists in the spaces between the mitochondrial membranes of retinal epithelial cells, splits into two molecules, gets activated, and enters the nucleus of the cell. In contrast, necroptosis is programmed necrosis within the cell, in which receptor-interacting protein kinase 1 (RIP1) and receptor-interacting protein kinase 3 (RIP3) form a complex and perform their functions [35]. In addition to activating AIF in retinal cells, blue light also stimulates RIP1 and RIP3 activation in retinal ganglion cells [36]. Osborne et al. (2017) demonstrated that while AIF was expressed intact in retinal cells cultured under dark conditions, it was expressed as two fragments under blue light conditions. Furthermore, retinal ganglion cells exposed to blue light reportedly showed a lower survival rate than those exposed to dark illumination, and the survival rate increased significantly when the expression of RIP1 and RIP3 proteins was inhibited through small interfering RNA (siRNA) technology [34]. It is evident that blue light activates both apoptosis and apoptotic necrosis in retinal cells, which may contribute to the onset or exacerbation of glaucoma.

## 4. Red Light and PBM Therapy

PBM is used, in its simplest form, for the biomodulation of photons of light. Among the various light wavelength ranges, red light has been observed to show a positive therapeutic effect [37,38,39]. In the past, lasers gained attention as a means of scanning red light; however, continuously scanning light at a wavelength of 620 nm could damage the eyes [39]. However, in recent years, LEDs have completely compensated for these disadvantages, and near-infrared rays with a wavelength range of approximately 780–940 nm have also become available [38]. PBM therapy was initially named low-level laser (light) therapy (LLLT), a biological treatment using a laser or LED, but was changed to PBM due to ambiguity in the standard of the low–level [37]. According to Calabrese et al. (2013) [40], no biological change is induced when red light intensity is low, and the ‘hormesis effect’ is observed above a certain intensity [41]. It has also been reported that when the light intensity exceeded the above-mentioned level, the positive effect decreased and tissue damage occurred [42].

Another study confirmed that treatment with a red light of appropriate intensity reduced oxidative stress and inflammation in living organisms, helping in the regeneration of cells and rapid tissue recovery [43,44]. Active studies on the influences of PBM therapy show positive effects on peripheral nerve tissue repair [45,46,47,48,49,50]. Peripheral nerve lesions have caused significant impairment in individuals’ daily lives due to the lack of effective treatments for recovery. At the cellular level, PBM therapy is associated with efficient nerve regeneration by improving nutritional status, reducing inflammation, and promoting the secretion of nerve factors [51,52]. In addition, changes in TNF-α, Il-1β, and GAP-43 levels suggest that PBM therapy in nerve injury is associated with the reduction of inflammatory cytokines and promotion of nerve regeneration [53,54]. Better wound healing in ischemic organs was also confirmed due to increased secretion of antiapoptotic factors [55,56]. Therefore, neurological PBM therapy provides faster and higher-quality recovery for the morphological healing of regenerating peripheral nerves and reduces inflammation and painful sensitivity [57,58]. This review was inspired by the positive effects of PBM therapy on these nerve cells and investigated its effects on the damage caused by light, especially glaucoma, which is directly related to the destruction of the optic nerve.

## 5. Mechanism of Eye Recovery through PBM Therapy

The therapeutic efficacy of red light is considered to be due to an increase in the activity of CCO, one of the complexes constituting the mitochondrial electron transport system [59,60]. CCO is an enzyme that catalyzes the oxidation reaction of cytochrome C, reducing oxygen molecules to water. It consists of two heme structures, cytochrome α and cytochrome α3, and two central copper structures, CuA and CuB [59]. According to a previous study by Mason et al. [61], a total of four electrons, transferred one by one through CCO, were delivered to the catalytic site (heme iron) with a binuclear copper center (cytochrome α3/CuB) through reduced cytochrome C, CuA, and cytochrome α. One molecule of oxygen is reduced at the catalytic site to form two molecules of water, and it has been reported that ATP synthesis is activated by the proton gradient [42,62]. Activation of CCO is greater than that of nitric oxide (nitric oxide: a gas that has a stronger affinity for CCO than oxygen and is structurally similar to oxygen), which functions as a competitive endogenous mediator of CCO when oxygen is enriched. It is induced by increasing the binding force to CCO [63,64]. However, Cleeter et al. (1994) confirmed that cytochrome C oxidase in the state of potential glaucoma has a low oxygen concentration, which decreases its activity and inhibits energy production [65]. In contrast, Brown and Cooper [66] found that red light plays a role in increasing the activity of CCO by photodissociating nitric oxide from cytochrome C oxidase. The PBM treatment is more effective since damaged cells and tissues contain more nitric oxide than healthy cells [42]. It has also been reported that when red light increases the activity of CCO, energy levels increase as ATP production improves and antioxidant production, such as of vitamins C and E, is stimulated, resulting in a decrease in ROS levels for a long period.

The increase in IOP transforms the eye into a state susceptible to secondary damage [67]. To prevent and treat glaucoma, IOP increase, and aging, the two fundamental causes, must be suppressed. As a result, PBM therapy is emerging as an effective means of enhancing mitochondrial function in retinal ganglion cells [43,44]. Osborne et al. (2017) artificially increased IOP in an experiment using rats. The control group was reared under dark conditions, and the experimental group was reared under red light (16.5 watts/m, 3000 lux, 625–635 nm) for a week. The results confirmed that the retinal epithelial cells of rats treated with red light had far less damage from IOP than those of the rats in the control group. Another study that compared and analyzed the effects of blue versus red light reported that in the presence of each of the lights of optimal energy value (blue light at 470 nm, 12.08 W/m2; red light at 630 nm, 6.5 W/m2), the survival rate of cells exposed to red light was higher than that of those exposed to blue light. The results of this study indicate that red light enhances mitochondrial function and increases ATP synthesis [20]. Further studies also proved that while blue light increases ROS, red light enhances mitochondrial function by regulating ROS. Furthermore, red light alleviates the damage caused by ischemia and reduces glial fibrillary acidic protein (GFAP), associated with the stress response. Therefore, blue light is damaging to retinal cells, while red light is beneficial [20].

## 6. Advantages of PBM Therapy

**Figure 3:**
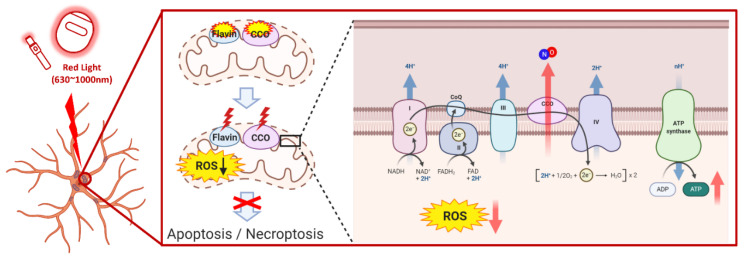
Illustration showing the proposed mechanism by which red light may have a beneficial effect on glaucoma. This figure was created using PowerPoint & BioRender (Available online: http://biorender.com (accessed on 9 April 2020).

The most common treatment for glaucoma is the prescription of eye drops. In addition to the hypotensive drugs, modern devices that inject drugs, locally and systemically, to lower IOP are also used. However, low patient compliance and unstable adherence are the major disadvantages of eye drop therapy. Regarding surgery, although traditional trabeculectomy remains the standard method of treatment, the latest trend is focused on improving the risk/benefit ratio of minimally invasive glaucoma surgery. This is because PBM is a safer and more efficient technique for lowering IOP than standard surgery. The red light (630–1000 nm) used for PBM therapy is a long-wavelength light that can pass through thick tissues compared to other wavelengths (Figure 3). Thus, it may be expected that retinal ganglion cells may now be treated non-surgically, without the need to cut the eye, through the therapeutic effects of red light. In addition, the fact that drug therapies were administered directly to the eyes increased the overall risk of side effects. However, red light is absorbed as it passes through the retina to induce therapeutic effects, so the possibility of side effects is relatively low [68].

## 7. Conclusions

The eye is one of the few major organs constantly exposed to the external environment. Various wavelengths of light from the outside environment continuously enter the retina and eventually affect the eye’s health. Based on the results of previous studies, it can be concluded that the increase in the incidence of glaucoma and the decrease in the age of onset may be attributed to the relationship between myopia and blue light and that the use of red light in promoting cell regeneration and reducing ROS can be a valid treatment strategy for this condition. Blue light, a short-wavelength visible light, induces apoptosis by disrupting the balance between mitochondrial antioxidative and ROS production processes in retinal ganglion cells; both these processes play an important role in visual processing (Figure 2). Owing to the deformation of the eyeball, a myopic eye is particularly susceptible to secondary damage caused by blue light. The most basic and effective measure to prevent such eye damage is to avoid excessive exposure to blue light. Basically, since glaucoma is caused by an increase in IOP or damage to the optic nerve, it is necessary to wear sunglasses when outdoors to prevent strong light from entering the eyeball and properly adjust the brightness of digital screens [69,70,71]. These habits are basic and routine methods recommended to be maintained even after glaucoma surgery [72]. However, treatment after the onset of glaucoma is still limited.

In contrast, PBM therapy, which is emerging as a new treatment for glaucoma, induces the inhibition of nitric oxide in the electron transport system and promotes an increase in the activity of CCO, reduces oxidative stress and inflammatory reactions in the eye, and increases energy production in the cells (Figure 3). Furthermore, since red light has a high tissue penetration rate and is a non-surgical treatment, it has fewer side effects and is less burdensome for the retina. However, the current treatment options for glaucoma are limited to direct injection of drugs into the eye as eye drops or laser ablation of the trabecular meshwork to reduce IOP. There is a need for additional epidemiological and clinical studies on both the harmful and the therapeutic effects of light on eye diseases. Currently, research is underway to develop eye treatment technologies and devices using the enhancing effect of red light [73], as well as to develop special lenses that would convert UV rays into green or red light [74]. Many positive effects are anticipated through the synergistic effects of the combination of PBM therapy, which enhances the resilience of the optic nerve, along with existing treatments. The general application of PBM treatment strategies for retinal diseases and glaucoma should be considered a necessary prospect for the future.

## Data Availability

Not applicable.
